# COVID-19 and its negative impact on the mental health of health professionals: an integrative literature review

**DOI:** 10.47626/1679-4435-2022-894

**Published:** 2022-03-30

**Authors:** Tácia Gabriela Vilar dos Santos Andrade, Ana Beatriz da Silva Feitosa, Laiana de Souza Silva, Nylene Maria Rodrigues da Silva

**Affiliations:** 1 Fisioterapia, Faculdade de Integração do Sertão (FIS), Serra Talhada, PE, Brazil.; 2 Medicina, Faculdade de Medicina de Olinda, Olinda, PE, Brazil.; 3 Medicina, Centro de Estudos Superiores de Maceió, Maceió, AL, Brazil.; 4 Fisioterapia e docência, FIS, Serra Talhada, PE, Brazil.

**Keywords:** professional burnout, pandemics, COVID-19

## Abstract

The current pandemic caused by the severe acute respiratory syndrome coronavirus 2 has triggered a scenario of danger and fear of contagion because of the elevated transmissibility and mortality. This in turn is responsible for development of anxieties and feelings of psychological suffering, triggering possible harm to the mental health of the health professionals who are daily faced with this battlefield scenario. This study aimed to assess the impact of the pandemic caused by COVID-19 on the mental health of health professionals working in this situation. An integrative literature review was conducted based on searches of the electronic scientific databases Virtual Health Library (Biblioteca Virtual em Saúde), National Library of Medicine, Physiotherapy Evidence Database, and Scientific Electronic Library Online. Publications were only identified in the Virtual Health Library database, with a total of 547 articles. After the filtering process, a total of 13 articles remained, which were screened by reading titles, abstracts, and full texts, leaving a total of eight articles, on which the manuscript is based. The scenario of uncertainties, anxieties, and fears faced by health professionals can have negative psychological repercussions for their health.

## Introduction

In December of 2019, the first cases of an infectious and contagious disease, were reported in Wuhan, in China. COVID-19 is caused by the severe acute respiratory syndrome coronavirus 2 (SARS-CoV-2).^1^ This disease has extremely high transmissibility and in March 2020 the World Health Organization (WHO) confirmed that there were cases of the disease in all continents of the world, characterizing a pandemic situation, and declared it a public health emergency.^2,3^

It is common for clinical manifestations to vary, ranging from asymptomatic through mild and severe cases, including the possibility of deaths if the situation deteriorates.^4,5^ COVID-19 can involve and compromise many of the body’s different systems, such as, for example, the muscular, gastrointestinal, nervous, and, primarily, respiratory systems, developing symptoms such as coughing, dyspnea, and the severe acute respiratory syndrome (SARS).^1^

*A priori*, contagion and transmission can occur through close and unprotected contact with contaminated people or materials. Because of this, it is necessary to take precautions when handling materials and to distance and isolate people.^6,7^ However, although these measures are necessary, there is a part of the population that needs to come into daily contact with people who are suspected of having or have been diagnosed with the disease: healthcare workers. This situation of danger and fear of contagion is responsible for triggering anxieties and feelings of psychological suffering, and can possibly cause harm to these professionals’ mental health.^8^

Thus, the widescale development of COVID-19 has subjected this subset of the population to a daily workload involving overload, fatigue, stress, and inadequate infrastructure, exposing them to long shifts and daily contact with death, in addition to the constant fear of contamination and transmission of the disease, which could prove fatal to their relatives.^8,9^

Worry and stress generated in the workplace is a common phenomenon and one that compromises professionals’ satisfaction and sense of achievement during their working days, especially when exposed to the risk factors mentioned above. This harm to mental health can manifest as a disease known as burnout syndrome, characterized by emotional exhaustion, reduced professional achievement and worker depersonalization.^10^ Thus, the objective of the present study was to assess the impact that the COVID-19 pandemic has had on the mental health of healthcare professionals working in this situation.

## Methods

An integrative literature review was conducted. This is a method for conducting a study on a specific subject based on previous studies and using evidence-based practices. The review was performed following the six phases proposed by Souza et al.:^11^ 1) definition of a guiding question; 2) searching or sampling the literature; 3) data collection; 4) critical analysis of the studies included; 5) discussion of the results; and 6) presentation of the integrative review.

### Definition of the guiding question

The review was initiated after the following question had been defined: “What impact has the COVID-19 pandemic had on the mental health of health professionals who have been working during this period?”.

### Searching or sampling the literature

The literature sample was obtained by searches conducted on the scientific electronic databases of the Virtual Health Library (BVS - Biblioteca Virtual em Saúde), the National Library of Medicine (PubMed), the Physiotherapy Evidence Database (PEDro), and the Scientific Electronic Library Online (SciELO). The search terms used to identify the studies were “Esgotamento profissional”, “Pandemias” and “COVID-2019”, selected from the BVS Descritores em Ciências da Saúde (DeCS), and “Professional burnout”, “Pandemics”, and “COVID-2019”, selected from the Medical Subject Headings (MeSH), combined using the Boolean operator “AND”.

After the search, the full list of articles was subjected to a filtering process. Initially, the “full text” filter was applied, the “healthcare workers” category was selected, and articles involving “diagnostic studies” that had been published from “January 2020 to June 2021” were sought.

The inclusion criteria adopted were full text primary articles published in Portuguese, English, or Spanish that covered the subject proposed. Studies were excluded if they were unrelated to the study proposal, were review articles, could not be accessed, or did not take adequate precautions to ensure patient integrity and safety.

### Data collection

Certain processes were performed to extract data from the articles selected, such as 1) identification of the article; 2) identification of the institution hosting the study; 3) identification of the type of publication; 4) analysis of the study’s methodological characteristics; and 5) assessment of methodological rigor.

### Critical analysis of the studies included

Critical analysis of the studies selected was performed independently by three reviewers, and a fourth assessor was consulted after the manuscript had been prepared. Evidence levels were classified according to the following levels: level I, level II, level III, level IV, level V, level VI, and level VII; where the strength of evidence is inversely proportional to the classification level.

### Discussion of results and presentation of the integrative review

The articles were discussed descriptively and the most important information they contain with relevance to understanding the subject was tabulated. Additional discussions that supplement the study data and confirm the theoretical framework were also presented.

## Results

Eligible publications were only identified in one of the database, the BVS, which returned a total of 551 articles. Application of the “full text” filter excluded 30 articles, application of the “healthcare workers” filter excluded 391 articles, and application of the “diagnostic study” filter excluded 117 articles. After the filtering process was complete, 13 articles remained and were screened by reading the titles, abstracts, and full texts, resulting in exclusion of five studies, leaving eight articles to make up the sample for the review.

**Figure 1:**
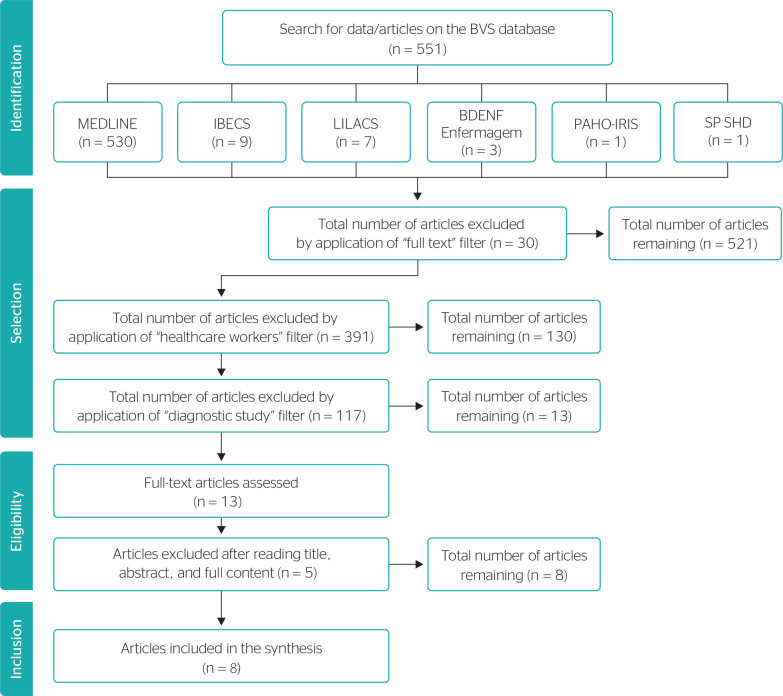
Flow diagram illustrating the quantitative process of identification, selection, eligibility, and inclusion of data and articles. BDENF Enfermagem = Nursing Database; BVS. = Biblioteca Virtual em Saúde; IBECS = Índice Bibliográfico Espanhol de Ciências de Saúde; LILACS = Literatura Latino-Americana e do Caribe em Ciências da Saúde; MEDLINE = Medical Literature Analysis and Retrieval System Online; PAHO-IRIS = SP SHD = São Paulo State Health Department.

Figure 1 contains a flow diagram illustrating the quantitative process of identification, selection, screening for eligibility, and inclusion of data from the articles found up to the final phase of filtering, representing the final sample of studies used for the review. Table 1 presents the qualitative characteristics of the publications selected for the review synthesis, listing author, year of publication, title, type of study, and objective. Table 2 presents qualitative characteristics related to the main results reported in the studies.

**Table 1:** Qualitative characteristics of publications selected for the review synthesis

Authors	Title of article	Type of study	Objective
Duarte et al.^12^	*Burnout* among Portuguese healthcare workers during the COVID-2019 pandemic	Cross-sectional study	To assess healthcare workers in terms of the contributions of sociodemographic mental health variables to three dimensions of burnout: personal, work-related, and client-related.
Firew et al.^13^	Protecting the front line: a cross-sectional survey analysis of the occupational factors contributing to healthcare workers’ infection and psychological distress during the COVID 19 pandemic in the USA	Cross-sectional study	To assess factors contributing to healthcare worker infection and psychological distress during the COVID-19 pandemic in the United States.
Hawari et al.^14^	The inevitability of COVID-2019 related distress among healthcare workers: findings from a low caseload country under lockdown	Cross-sectional study	To characterize psychological distress and factors associated with distress in healthcare professionals working during a rigorous lockdown in a country in the era of Covid-19.
Lai et al.^15^	Factors associated with mental health outcomes among healthcare workers exposed to coronavirus disease 2019	Cross-sectional study	To assess the magnitude of mental health outcomes and associated factors among health care workers treating patients exposed to COVID-19 in China.
Li et al.^16^	Vicarious traumatization in the general public, members, and non-members of medical teams aiding in COVID-2019 control	Descriptive study	Information not provided.
Luceño-Moreno et al.^17^	Symptoms of posttraumatic stress, anxiety, depression, levels of resilience and burnout in Spanish health personnel during the COVID-2019 pandemic	Cross-sectional study	Analyze posttraumatic stress, anxiety, and depression during the COVID-19 pandemic.
Matsuo et al.^18^	Prevalence of healthcare worker burnout during the coronavirus disease 2019 (COVID-2019) pandemic in Japan	Cross-sectional study	To evaluate the prevalence of burnout among frontline healthcare workers during the COVID-19 pandemic in Japan based on careers and other factors.
Sunjaya et al.^19^	Depressive, anxiety, and burnout symptoms on healthcare personnel at a month after COVID-2019 outbreak in Indonesia	Cross-sectional study	To explore depressive, anxiety, and burnout symptoms among health professionals with higher risk for psychological trauma.

**Table 2:** Qualitative description of the main results in the manuscripts used for this review

Authors	Main results
Duarte et al.^12^	High levels of burnout were found in 1,055 professionals (52.5%) and high work-related burnout was found in 1,066 (53.1%). Depression (70.6%) and stress (63.4%) were also reported in the majority of participants.
Firew et al.^13^	The majority reported taking preventative measures to protect the people with whom they lived, including all of the requirements for home precautions (56.96%), isolation (41.39%), moving to a different residence temporarily (12.09%), or sending cohabitants away from home (7.27%). Isolation and living alone were associated with significantly higher levels of depressive symptoms. Isolation, moving into a different residence, and taking necessary precautions at home while continuing to live with cohabitants were associated with elevated anxiety symptoms.
Hawari et al.^14^	After administration of a questionnaire developed by a core team of medical staff involved in Covid-19 research and screening, it was observed that 20% of the sample suffered from very severe distress and 32% reported high levels of distress. Approximately 34% and 19% reported at least moderate anxiety and depression, respectively. Additionally, 34.3% of practitioners reported exhaustion; and 28.6% reported having sleep issues (trouble falling asleep or staying up at least half the night). Of those 28.6% reporting sleep-related issues, 55.6% experienced problems functioning during the day because of these.
Lai et al.^15^	A considerable proportion of health professionals reported symptoms of harm to mental health. Of the total sample, 634 had depression (50.4%), 560 reported anxiety (44.6%), 427 reported insomnia (34%), and 899 reported distress (71.5%).
Li et al.^16^	Around 139 health professionals exhibited traumatization by the virus (loss of appetite, fatigue, physical decline, sleep disorders, irritability, inattention, fear, and despair), while 103 reported psychological traumas (fear of contact with the public), 28 reported behavioral changes (irritability, restlessness) and 34 developed emotional responses (lack of emotional resistance).
Luceño-Moreno et al.^17^	Around 833 (58.6%) individuals exhibited mild anxiety disorder, 295 (20.7%), had severe anxiety, 648 (46%) had mild depression, 82 (5.3%) had severe depression, 375 (26.4%) had moderate posttraumatic stress, and 805 (56.6%) had severe psychiatric disorders. Additionally, medium professional exhaustion was exhibited by 328 (23.1%) of the participants and high exhaustion was manifest by 584 (41%).
Matsuo et al.^18^	The overall burnout prevalence was 31.4% (98 of 312). Of 126 nurses, 59 (46.8%) were experiencing burnout; of 22 radiology technicians, 8 (36.4%) were experiencing burnout; and of 19 pharmacists, 7 (36.8%) were experiencing burnout.
Sunjaya et al.^19^	Around 22.8% of the healthcare personnel experienced depressive symptoms, 28.1%, anxiety, and 26.8% burnout.

## Discussion

Recent studies have demonstrated that the COVID-19 pandemic caused an unprecedented crisis in more than 200 countries and healthcare professionals undoubtedly constitute one of the classes most affected.^20^ This population subset has been the most affected psychologically because of many different daily stressors, such as increased workloads, fear of contaminating families, and of becoming contaminated oneself, and lack of government investment and support. Moreover, the high numbers of sick and dying people during the pandemic imposes a high risk of occupational psychosocial harm on teams working on the frontline.^21^

One of the studies, conducted with physicians from Wuhan, revealed that they were under enormous pressure, including high risk of infection and inadequate protection against contamination, excessive workloads, frustration, discrimination, isolation, caring for patients with negative emotions, lack of contact with families, and exhaustion.^22^ Concomitantly, this excessive workload appears to facilitate mental and physical sickness in healthcare workers, in addition to increasing the likelihood of absenteeism, workplace accidents, medication errors, and work overload.^21^

Fear of being infected, proximity to suffering patients, or their death, family-members’ anguish linked to shortages of medical supplies, uncertain information about many resources, loneliness, and worry about loved ones were other elements that also contributed to psychological suffering and mental sickness among health professionals, leading some to no longer wish to work.^22,23^

Although healthcare professionals are used to dealing with situations involving psychological tension, the COVID-19 pandemic provoked a different scenario in which shortages of personal protective equipment, ventilators, and medications to treat critical patients, shortages of intensive care beds, and unpredictability of working conditions, all compounded by the long working hours and deaths of team members, contribute even more to harming their mental health.^20^

The result was that this situation caused mental health problems such as burnout syndrome, triggering symptoms such as stress, anxiety, depressive symptoms, insomnia, negation, rage, and fear, which are problems that not only affect attention, understanding, and decision-making ability, but which can also have lasting effects on overall wellbeing, since self-care is restricted and leisure ever more scarce.^22^

Burnout syndrome, or professional exhaustion syndrome has been added to the International Classification of Diseases (ICD) under code QD85. It can be defined as a prolonged emotional response provoked by chronic stressors in three categories: reduced sense of personal achievement, emotional exhaustion, and depersonalization.^24^

The reduction in personal sense of achievement is correlated to the fact that affected people make negative self-assessments of their own productivity and competencies. This situation can take a negative course in their working life, triggering reductions in the quality of service provided and in the professional’s self-esteem. Because of this, it is common that sufferers experience periods of dissatisfaction and malaise in the workplace, facilitating occurrence of undesirable events such as occupational accidents, social disengagement, and quitting work.^25,26^

Emotional exhaustion is related to a feeling of exhaustion and overload in emotional conditions, manifest as a lack of energy and an absence of the desire to perform the common tasks required of one’s professional practice, in addition to also affecting personal relations negatively. This factor can be considered the most marked and easiest to identify manifestation of the syndrome, since frustration exhibited by professionals manifests in a highly significant manner.^27,28^

Depersonalization emerges as an attempt to cope with the attrition that the person is suffering. It is common to develop symptoms that do not fit the professional’s habitual personality; generally leading to reduced empathy and difficulty with feeling sadness about patients’ suffering or even when the disease kills them. Moreover, there is increased indifference to the activities conducted by other professionals and personal and professional deconstruction becomes clear.^29^

The pandemic scenario led to an increasingly proactive role for healthcare workers as a class. This demanded that the profession reformulated its skills and molded itself to fit the needs of the healthcare system and the population itself. However, this situation of exposure and pressure can contribute to triggering the more severe manifestations of burnout syndrome.^30^

According to Ramírez-Ortiz et al.,^31^ comparing the periods before and during the pandemic, it is possible to observe an increase in the numbers of cases of burnout syndrome in health professionals after the start of the outbreak. This situation could mark the start of a larger problem in the future, when economic activity resumes and the pandemic ends.^31^

In view of these factors, mental health promotion has become a focus in hospital settings. The perception that a mental health deficit has a negative impact on productivity and on performance of professional activities has come to the attention of the managers of healthcare institutions. However, it can be observed that many health professionals remain resistant to the practice, refusing preventative and continuous treatment. It is therefore necessary to maintain this population under observation and analyze each member’s individual characteristics, in order to identify more satisfactory intervention strategies.^32,33^

## Conclusions

The pandemic has greatly intensified the role health professionals are expected to play, and situations involving great pressure and demand have developed because of this. The situation of uncertainties, anxieties, and fears that is being faced by the members of this profession is responsible for psychological repercussions that are negative for their health. The studies included in this review revealed increases in burnout syndrome related to extreme workloads, stress, anxiety, depression, and uncertainties caused by the direct and daily battle against COVID-19.

This study should provoke reflection on the need for mental health prevention and promotion in hospital settings. It should be emphasized that there is a need to prioritize care for these professionals and strategies should be implemented, such as protocols for psychological monitoring and shorter shifts, to enable greater opportunities for release from this critical environment. By so doing, the quality and safety of the service provided by these professionals can be ensured in a more satisfactory and healthy manner.
